# Supplementary material for: Meta-taxonomic analysis of prokaryotic and eukaryotic gut flora in stool samples from visceral leishmaniasis cases and endemic controls in Bihar State India
Source: PLoS Negl Trop Dis. 2019 Sep 6;13(9):e0007444. doi: 10.1371/journal.pntd.0007444 (PMC6750594; doi:10.1371/journal.pntd.0007444)
Supplement: S1 Checklist — Provides details of how our study complies with guidelines for epidemiological case-control studies set down by the STROBE consortium of international epidemiologists, methodologists, statisticians, researchers and journal editors. (PDF) [file pntd.0007444.s001.pdf]

S1 Checklist

Meta-taxonomic Analysis of Prokaryotic and Eukaryotic Gut Flora in Stool Samples from Visceral Leishmaniasis Cases and Endemic Controls in Bihar State India

Rachael Lappan,[1] Cajsa Classon,[2] Shashi Kumar,[3] Om-Prakash Singh,[3] Ricardo V. de Almeida,[4] Jaya Chakravarty,[3] Poonam Kumari,[3] Sangeeta Kansal,[3] Shyam Sundar[3,*] and Jenefer M. Blackwell[1,5,*,§]

[1]Telethon Kids Institute, The University of Western Australia, Nedlands, Western Australia, Australia

[2]Department of Microbiology, Tumor and Cell Biology, Karolinska Institutet, Solna, Sweden

[3]Department of Medicine, Institute of Medical Sciences, Banaras Hindu University, Varanasi, India

4Departamento de Bioquímica, Centro de Biociências, Universidade Federal do Rio Grande do Norte, Natal, Brazil

[5]Department of Pathology, University of Cambridge, Cambridge, UK

[§]Corresponding Author: Professor Jenefer Blackwell, Telethon Kids Institute, PO Box 855, West Perth, Western Australia 6872, Australia. E-mail: jenefer.blackwell@telethonkids.org.au

[*] Equal senior authors

This is a scoping study that uses 16S rRNA and 18S rRNA meta-taxonomic analysis of fecal samples from 23 visceral leishmaniasis cases and 23 healthy endemic controls from Bihar State in India. The primary aim was an observational study to determine the composition of gut prokaryotic and eukaryotic microflora in this region of India endemic for VL, with a small case-control comparison to determine whether this microflora differs between VL cases and non-VL endemic controls. We compare microscopic determination of helminth infections with 18S rRNA data, and report on secondary aims of determining the potential influence of prokaryotic or eukaryotic taxa known to contain pathogenic species on gut microbial diversity. The table below provides the line numbers in the manuscript that address items on the STROBE checklist [1]. **Note:** Due to the nature of our study, many of the criteria are not relevant to our type of study.

STROBE Statement—Checklist of items that should be included in reports of ***case-control studies***

	Item No	Recommendation
**Title and abstract**	1	(*a*) Indicate the study's design with a commonly used term in the title or the abstract Case-Control used in title lines 1-3.
		(*b*) Provide in the abstract an informative and balanced summary of what was done and what was found See abstract lines 39-59.
**Introduction**		
Background/rationale	2	Explain the scientific background and rationale for the investigation being reported Introduction lines 118-144.
Objectives	3	State specific objectives, including any prespecified hypotheses Introduction lines 137-144.
**Methods**		
Study design	4	Present key elements of study design early in the paper Case-control selection lines 150-157.
Setting	5	Describe the setting, locations, and relevant dates, including periods of recruitment, exposure, follow-up, and data collection Lines 149-157
Participants	6	(*a*) Give the eligibility criteria, and the sources and methods of case ascertainment and control selection. Give the rationale for the choice of cases and controls Lines 164-167.
		(*b*) For matched studies, give matching criteria and the number of controls per case Lines 154-156.
Variables	7	Clearly define all outcomes, exposures, predictors, potential confounders, and effect modifiers. Give diagnostic criteria, if applicable Lines 150-157.
Data sources/ measurement	8*	For each variable of interest, give sources of data and details of methods of assessment (measurement). Describe comparability of assessment methods if there is more than one group Lines Lines 150-157; 164-167; 177-183; 186-197; 200-214; 217-234; 237-258; 261-279.
Bias	9	Describe any efforts to address potential sources of bias
Study size	10	Explain how the study size was arrived at - Small scoping study; samples sizes equivalent to similar published studies for microbiome data.
Quantitative variables	11	Explain how quantitative variables were handled in the analyses. If applicable, describe which groupings were chosen and why. Data for age analysed on a continuous scale .
Statistical methods	12	(*a*) Describe all statistical methods, including those used to control for confounding
		(*b*) Describe any methods used to examine subgroups and interactions
		(*c*) Explain how missing data were addressed
		(*d*) If applicable, explain how matching of cases and controls was addressed
		(*e*) Describe any sensitivity analyses All covered under lines 237-279 of methods.
**Results**		
Participants	13*	(a) Report numbers of individuals at each stage of study—eg numbers potentially eligible, examined for eligibility, confirmed eligible, included in the study, completing follow-up, and analysed. Single time point of collection of samples; sample size stayed the same as recruitment size.
		(b) Give reasons for non-participation at each stage There we no non-participants.
		(c) Consider use of a flow diagram

Descriptive data	14*	(a) Give characteristics of study participants (eg demographic, clinical, social) and information on exposures and potential confounders. Provided in the methods section.
		(b) Indicate number of participants with missing data for each variable of interest. See lines 306-309 for numbers of individuals not take forward in part of the analysis due to low sequence reads.
Outcome data	15*	Report numbers in each exposure category, or summary measures of exposure. Results provide details of results of sequence analysis for different groups, and numbers contributing as indicated under point 14 above.
Main results	16	(*a*) Give unadjusted estimates and, if applicable, confounder-adjusted estimates and their precision (eg, 95% confidence interval). Make clear which confounders were adjusted for and why they were included. Not applicable to our kind of data analysis.
		(*b*) Report category boundaries when continuous variables were categorized. Not applicable to our kind of analysis.
		(*c*) If relevant, consider translating estimates of relative risk into absolute risk for a meaningful time period. Not relevant to our study.
Other analyses	17	Report other analyses done—eg analyses of subgroups and interactions, and sensitivity analyses This is not relevant to our study analysis. We provided detailed description of the analysis of sequence data for microbes in the gut of cases and controls.
**Discussion (lines 497-643)**		
Key results	18	Summarise key results with reference to study objectives. We described the microbiomes of individuals living in this region of India. We provided information on interesting differences in gut microbes for VL cases compared to endemic controls.
Limitations	19	Discuss limitations of the study, taking into account sources of potential bias or imprecision. Discuss both direction and magnitude of any potential bias. This was a scoping study that demonstrates the value of the technology employed, and indicates where further larger scale studies would be required.
Interpretation	20	Give a cautious overall interpretation of results considering objectives, limitations, multiplicity of analyses, results from similar studies, and other relevant evidence. We have been cautious in the interpretation of our data.
Generalisability	21	Discuss the generalisability (external validity) of the study results. We have discussed the applicability of our methods to the study of NTDs generally.
**Other information**		
Funding	22	Give the source of funding and the role of the funders for the present study and, if applicable, for the original study on which the present article is based. We have provided the information on funding.

*Give information separately for cases and controls.

Reference

1. von Elm E, Altman DG, Egger M, Pocock SJ, Gotzsche PC, Vandenbroucke JP, et al. The Strengthening the Reporting of Observational Studies in Epidemiology (STROBE) statement: guidelines for reporting observational studies. PLoS medicine. 2007;4(10):e296. doi: 10.1371/journal.pmed.0040296. PubMed PMID: 17941714.
