# Supplementary material for: Meta-taxonomic analysis of prokaryotic and eukaryotic gut flora in stool samples from visceral leishmaniasis cases and endemic controls in Bihar State India
Source: PLoS Negl Trop Dis. 2019 Sep 6;13(9):e0007444. doi: 10.1371/journal.pntd.0007444 (PMC6750594; doi:10.1371/journal.pntd.0007444)
Supplement: S1 Table — (PDF) [file pntd.0007444.s002.pdf]

S1 Table. Metadata for endemic control (EC) and visceral leishmaniasis (VL) ndividuals contributing to the study.

SampleID	Group	Sex	Age	Village	Block/PHC	District	Date Collect	Patient Type	Ascaris	Trichuris trichura	Hookworm	Hymenolepis	Pathogenic Helminth	>1 Pathogenic Helminth	Any Helminth	Filaria
EC1	EC	F	42	Jagarnathpur Doghra	SARAIYA	Muzaffarpur	24-May-17	Attendant EC	No	No	No	No	No	No	No	Negative
EC2	EC	F	7	Khajuri	SAHEBGANJ	Muzaffarpur	29-May-17	Field EC	No	No	No	Yes	No	No	Yes	Not tested
EC3	EC	F	11	Khajuri	SAHEBGANJ	Muzaffarpur	29-May-17	Field EC	Yes	No	No	No	Yes	No	Yes	Not tested
EC4	EC	M	20	Kerma	KURHANI	Muzaffarpur	24-May-17	Attendant EC	No	No	Yes	No	Yes	No	Yes	Negative
EC5	EC	F	40	Shahwajpur	KANTI	Muzaffarpur	24-May-17	Attendant EC	No	**Yes**	Yes	No	Yes	Yes	Yes	Positive
EC6	EC	F	40	Hiramma	TARIYANI	Sheohar	23-May-17	Attendant EC	Yes	Yes	No	No	Yes	Yes	Yes	Positive
EC7	EC	F	40	Rajwara	MUSHAHARI	Muzaffarpur	05-Oct-17	Attendant EC	NA	NA	NA	NA	NA	NA	NA	Not tested
EC8	EC	F	30	Andaul Urf BishunpurMandal	BARURAJ (MOTIPUR)	Muzaffarpur	18-May-17	Attendant EC	No	Yes	No	No	Yes	No	Yes	Negative
EC9	EC	F	42	Ghosaut	MINAPUR	Muzaffarpur	06-May-17	Attendant EC	No	No	No	No	No	No	No	Not tested
EC10	EC	F	35	Dumri Parmanandpur	BOCHAHA	Muzaffarpur	08-May-17	Attendant EC	No	No	Yes	No	Yes	No	Yes	Not tested
EC11	EC	M	18	Khajuri	SAHEBGANJ	Muzaffarpur	29-May-17	Field EC	No	No	No	No	No	No	No	Not tested
EC12	EC	F	45	Bajar Munria	MINAPUR	Muzaffarpur	25-May-17	Attendant EC	No	No	No	No	No	No	No	Negative
EC13	EC	F	40	Chhitri	SARAIYA	Muzaffarpur	04-Oct-17	Attendant EC	NA	NA	NA	NA	NA	NA	NA	Not tested
EC14	EC	M	19	Sahila Rampur	BOCHAHA	Muzaffarpur	26-May-17	Attendant EC	No	No	No	No	No	No	No	Negative
EC15	EC	M	25	Mahesha Farrukhpur	RUNNI SAIDPUR	Sitamarhi	16-May-17	Attendant EC	No	No	No	No	No	No	No	Negative
EC16	EC	F	35	Kamla Balia	BOCHAHA	Muzaffarpur	07-Oct-17	Attendant EC	NA	NA	NA	NA	NA	NA	NA	Not tested
EC17	EC	F	47	Makundpur	TARAIYA	Saran	10-May-17	Attendant EC	Yes	No	Yes	No	Yes	Yes	Yes	Not tested
EC18	EC	M	13	Khajuri	SAHEBGANJ	Muzaffarpur	29-May-17	Field EC	No	No	No	Yes	No	No	Yes	Not tested
EC19	EC	F	12	Khajuri	SAHEBGANJ	Muzaffarpur	29-May-17	Field EC	No	No	No	No	No	No	No	Not tested
EC20	EC	M	14	Khajuri	SAHEBGANJ	Muzaffarpur	29-May-17	Field EC	No	No	No	Yes	No	No	Yes	Not tested
EC21	EC	F	30	Singwari	KATRA	Muzaffarpur	06-May-17	Attendant EC	No	No	No	No	No	No	No	Negative
EC22	EC	F	12	Khajuri	SAHEBGANJ	Muzaffarpur	29-May-17	Field EC	No	No	No	Yes	No	No	Yes	Not tested
EC23	EC	F	40	Kenaru	KURHANI	Muzaffarpur	08-May-17	Attendant EC	No	No	No	No	No	No	No	Not tested
VL1	VL	M	20	Jagarnathpur Doghra	SARAIYA	Muzaffarpur	26-May-17	VL	No	No	No	No	No	No	No	Negative
VL2	VL	F	36	Sahila Rampur	BOCHAHA	Muzaffarpur	26-May-17	VL	No	No	No	No	No	No	No	Negative
VL3	VL	F	55	Madhopur Hazari	SAHEBGANJ	Muzaffarpur	31-May-17	VL	No	No	No	No	No	No	No	Positive
VL4	VL	F	9	Singwari	KATRA	Muzaffarpur	07-May-17	VL	No	No	No	No	No	No	No	Negative
VL5	VL	F	20	Shahbazpur	KANTI	Muzaffarpur	24-May-17	VL	No	Yes	Yes	No	Yes	Yes	Yes	Positive
VL6	VL	F	16	Madhopur	MADHUBAN	Purba Champaran	31-May-17	VL	No	No	Yes	No	Yes	No	Yes	Positive
VL7	VL	F	27	NA		NA	11-Oct-17	VL	NA	NA	NA	NA	NA	NA	NA	NA
VL8	VL	F	40	Kerma	KURHANI	Muzaffarpur	24-May-17	VL	No	No	No	No	No	No	No	Positive
VL9	VL	M	5	Madhopur	SAHEBGANJ	Muzaffarpur	31-May-17	VL	No	Yes	No	No	Yes	No	Yes	Negative
VL10	VL	M	10	Kamla Balia	BOCHAHA	Muzaffarpur	07-Oct-17	VL	NA	NA	NA	NA	NA	NA	NA	NA
VL11	VL	M	15	Hiramma	TARIYANI	Sheohar	23-May-17	VL	No	Yes	No	No	Yes	No	Yes	Negative
VL12	VL	F	25	Mohanpur	KURHANI	Muzaffarpur	04-Jun-17	VL	No	No	No	No	No	No	No	NA
VL13	VL	F	5	Basant Kharauna	KURHANI	Muzaffarpur	07-Oct-17	VL	NA	NA	NA	NA	NA	NA	NA	NA
VL14	VL	F	10	Bajar Munria	MINAPUR	Muzaffarpur	25-May-17	VL	No	No	Yes	No	Yes	No	Yes	Negative
VL15	VL	F	12	Mithepur Bin Toli	GARKHA	Saran	08-Oct-17	VL	NA	NA	NA	NA	NA	NA	NA	NA
VL16	VL	F	8	Andaul Urf BishunpurMandal	BARURAJ (MOTIPUR)	Muzaffarpur	18-May-17	VL	No	Yes	No	No	Yes	No	Yes	Positive
VL17	VL	F	40	Bishunpur Urf Bishunpur Anant	RUNNI SAIDPUR	Sitamarhi	31-May-17	VL	No	Yes	No	No	Yes	No	Yes	NA
VL18	VL	F	13	Chhitri	SARAIYA	Muzaffarpur	04-Oct-17	VL	NA	NA	NA	NA	NA	NA	NA	NA
VL19	VL	M	10	Rajwara	MUSHAHARI	Muzaffarpur	05-Oct-17	VL	NA	NA	NA	NA	NA	NA	NA	NA
VL20	VL	F	17	Gausara	KURHANI	Muzaffarpur	31-May-17	VL	No	No	No	No	No	No	No	Negative
VL21	VL	M	50	Ghosaut	MINAPUR	Muzaffarpur	05-May-17	VL	No	No	No	No	No	No	No	NA
VL22	VL	M	7	Madhopur Hazari	SAHEBGANJ	Muzaffarpur	31-May-17	VL	No	No	No	No	No	No	No	Positive
VL23	VL	F	45	MANIKPUR	SARAIYA	Muzaffarpur	17-Oct-17	VL	NA	NA	NA	NA	NA	NA	NA	NA
