# Supplementary material for: Meta-taxonomic analysis of prokaryotic and eukaryotic gut flora in stool samples from visceral leishmaniasis cases and endemic controls in Bihar State India
Source: PLoS Negl Trop Dis. 2019 Sep 6;13(9):e0007444. doi: 10.1371/journal.pntd.0007444 (PMC6750594; doi:10.1371/journal.pntd.0007444)
Supplement: S1 Fig — (PDF) [file pntd.0007444.s005.pdf]

S1 Figure

a)

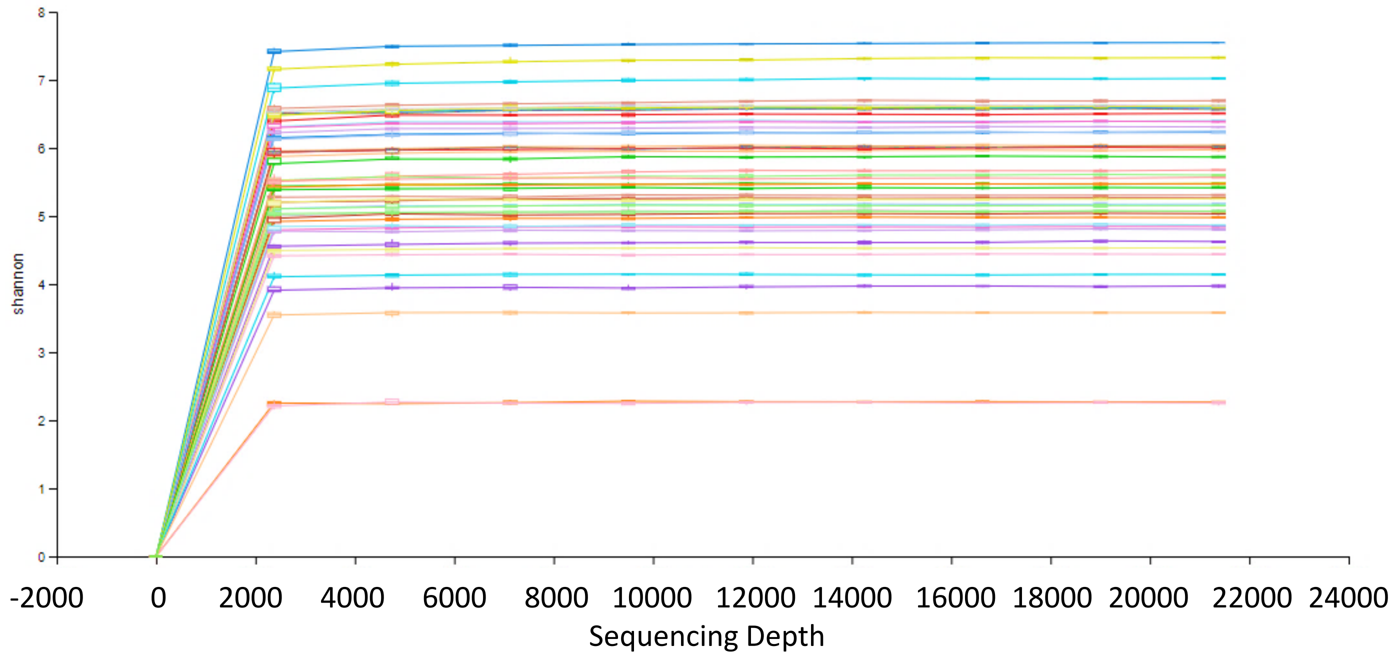

b)

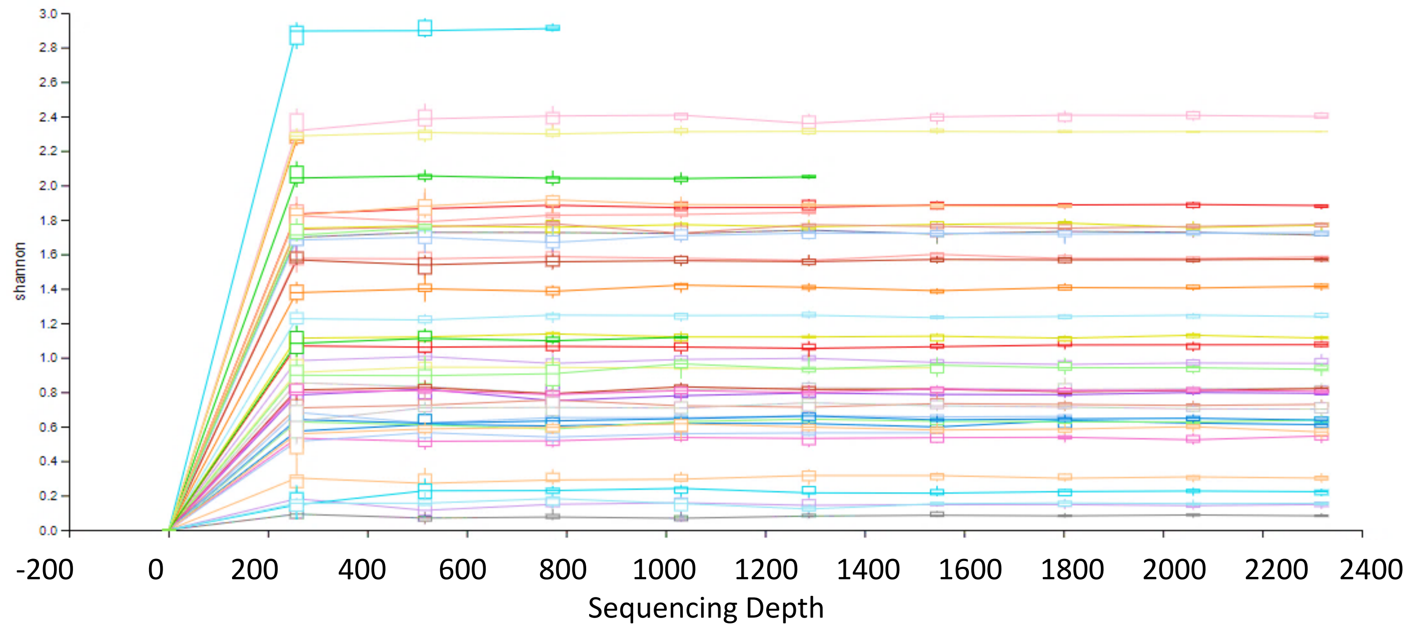

S1 Figure. Rarefaction plots based on Shannon's phylogenetic alpha diversity index for (a) 16S rRNA sequence date and (b) 18S rRNA sequence data (after filtering out contaminating bacterial sequences).
