# Supplementary material for: Meta-taxonomic analysis of prokaryotic and eukaryotic gut flora in stool samples from visceral leishmaniasis cases and endemic controls in Bihar State India
Source: PLoS Negl Trop Dis. 2019 Sep 6;13(9):e0007444. doi: 10.1371/journal.pntd.0007444 (PMC6750594; doi:10.1371/journal.pntd.0007444)
Supplement: S2 Fig — Taxa ordered by relative abundance of the contaminant “Eukaryota”. (a) VL case and EC samples. (b) Negative (MD labels) and positive (MOCK labels) controls. Negative controls indicate a small degree of laboratory cross-contamination of taxa identified in experimental samples. (c) Bar plot for 16S taxa that were contaminants in the negative control samples, colour key to relative abundances as for main Fig 1(a). Note that these percentages are relative to the much smaller number of processed reads for negative controls (9573±9294 compared to 71,123±22,122 for positive mock controls and 91,923±69,706 for VL cases and EC samples). The diversity within the samples was equivalent to VL cases and EC, indicating that they were likely due to cross-contamination in the endemic laboratory. The absolute numbers of reads were unlikely to have influenced the data and conclusions drawn for experimental samples. (PDF) [file pntd.0007444.s006.pdf]

S2 Figure

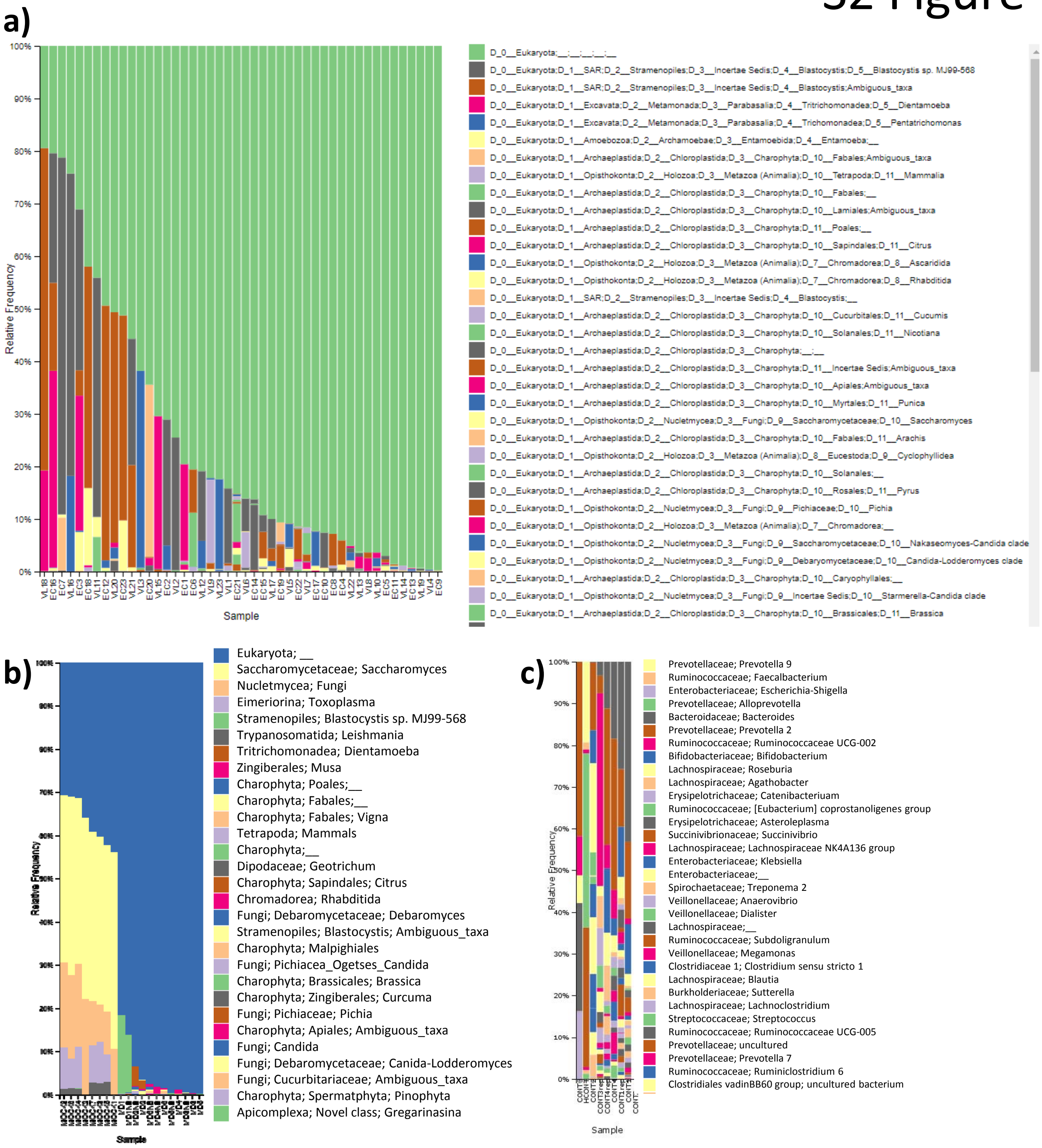

S2 Figure. (a) and (b) Bar plots for relative abundance of taxa for 18S rRNA data prior to filtering out contaminating bacterial species identified as unclassified "Eukaryota". Taxa ordered by relative abundance of the contaminant "Eukaryota". (a) VL case and EC samples. (b) Negative (MD labels) and positive (MOCK labels) controls. Negative controls indicate a small degree of laboratory cross-contamination of taxa identified in experimental samples. (c) Bar plot for 16S taxa that were contaminants in the negative control samples, colour key to relative abundances as for main figure 1(a). Note that these percentages are relative to the much smaller number of processed reads for negative controls (9573±9294 compared to 71,123±22,122 for positive mock controls and 91,923±69,706 for VL cases and EC samples). The diversity within the samples was equivalent to VL cases and EC, indicating that they were likely due to cross-contamination in the endemic laboratory. The absolute numbers of reads were unlikely to have influenced the data and conclusions drawn for experimental samples.
