# Supplementary material for: Meta-taxonomic analysis of prokaryotic and eukaryotic gut flora in stool samples from visceral leishmaniasis cases and endemic controls in Bihar State India
Source: PLoS Negl Trop Dis. 2019 Sep 6;13(9):e0007444. doi: 10.1371/journal.pntd.0007444 (PMC6750594; doi:10.1371/journal.pntd.0007444)
Supplement: S4 Fig — (a) and (b) bar plots for relative abundance of taxa by age and sex, respectively; colour key to relative abundances as for main Fig 1(a). (c) to (f) comparisons of species richness (number of ASVs) and alpha diversity measures (as labelled). (g) PCoA plots (left to right: PC1xPC2; PC1xPC3; PC2xPC3) for weighted UniFrac beta diversity by gender (see key). (h) Three-dimensional PCoA plot for weighted UniFrac beta diversity by age (see key for continuous scale). (PDF) [file pntd.0007444.s008.pdf]

S4 Figure

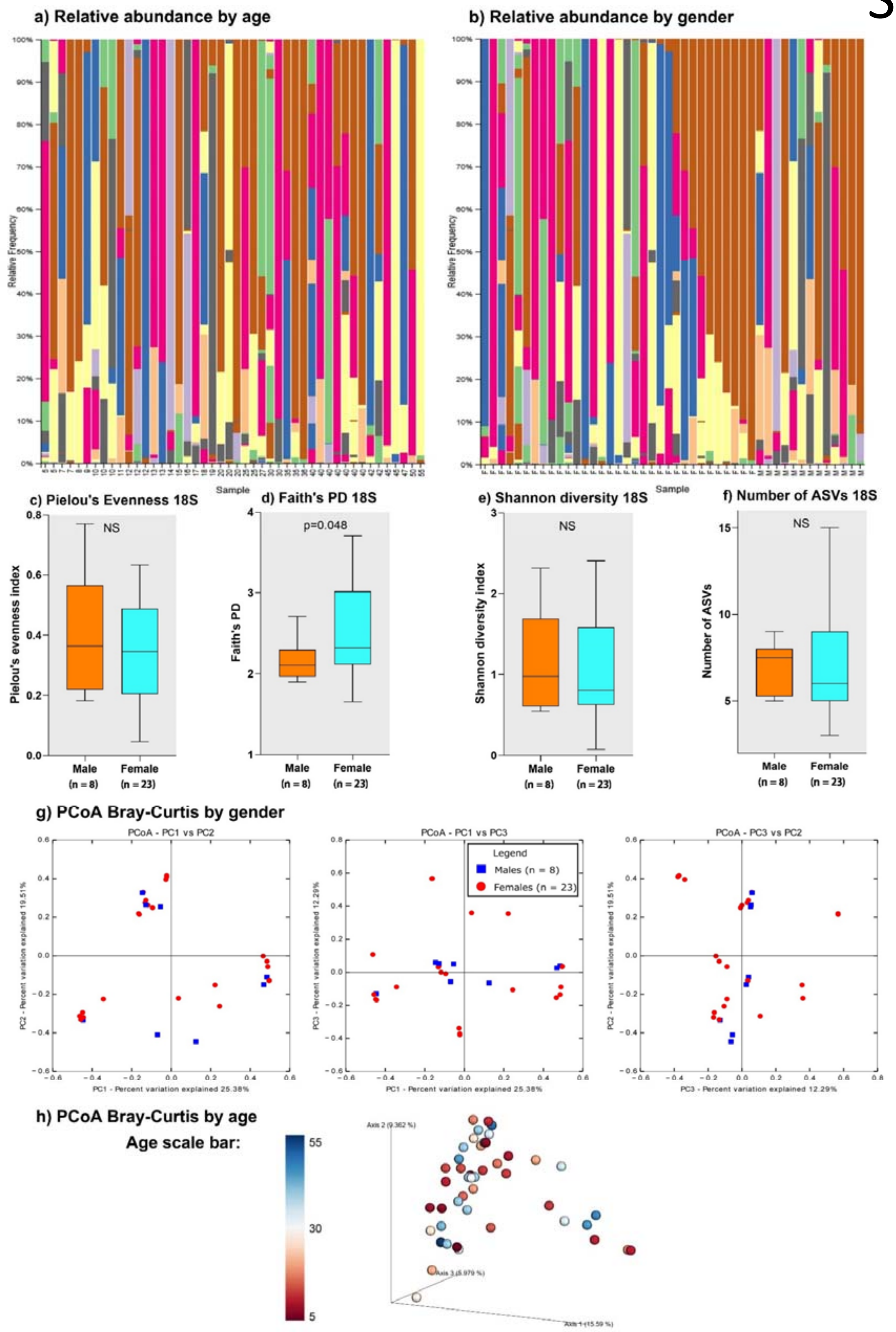

S4 Figure. Influence of age and gender on 18S rRNA-determined eukaryotic microbial profiles. (a) and (b) bar plots for relative abundance of taxa by age and sex, respectively; colour key to relative abundances as for main figure 1(a). (c) to (f) comparisons of species richness (number of ASVs) and alpha diversity measures (as labelled). (g) PCoA plots (left to right: PC1xPC2; PC1xPC3; PC2xPC3) for weighted UniFrac beta diversity by gender (see key). (h) Three-dimensional PCoA plot for weighted UniFrac beta diversity by age (see key for continuous scale).
