# Supplementary material for: Meta-taxonomic analysis of prokaryotic and eukaryotic gut flora in stool samples from visceral leishmaniasis cases and endemic controls in Bihar State India
Source: PLoS Negl Trop Dis. 2019 Sep 6;13(9):e0007444. doi: 10.1371/journal.pntd.0007444 (PMC6750594; doi:10.1371/journal.pntd.0007444)
Supplement: S6 Fig — (a) map of the district of Muzaffarpur showing subdistricts or block, annotated with the numbers of samples collected from different blocks. Listed below the map are numbers of samples from blocks outside of Muzaffarpur. (b) bar plots for relative abundance of taxa by district; colour key to relative abundances of taxa as for main Fig 2(a). (c) to (f) comparisons of species richness (number of ASVs) and alpha diversity measures (as labelled) by blocks with N≥3. (g) PCoA plots (left to right: PC1xPC2; PC1xPC3; PC2xPC3) for Bray’s dissimilarity beta diversity by colour-coded blocks. (PDF) [file pntd.0007444.s010.pdf]

S6 Figure

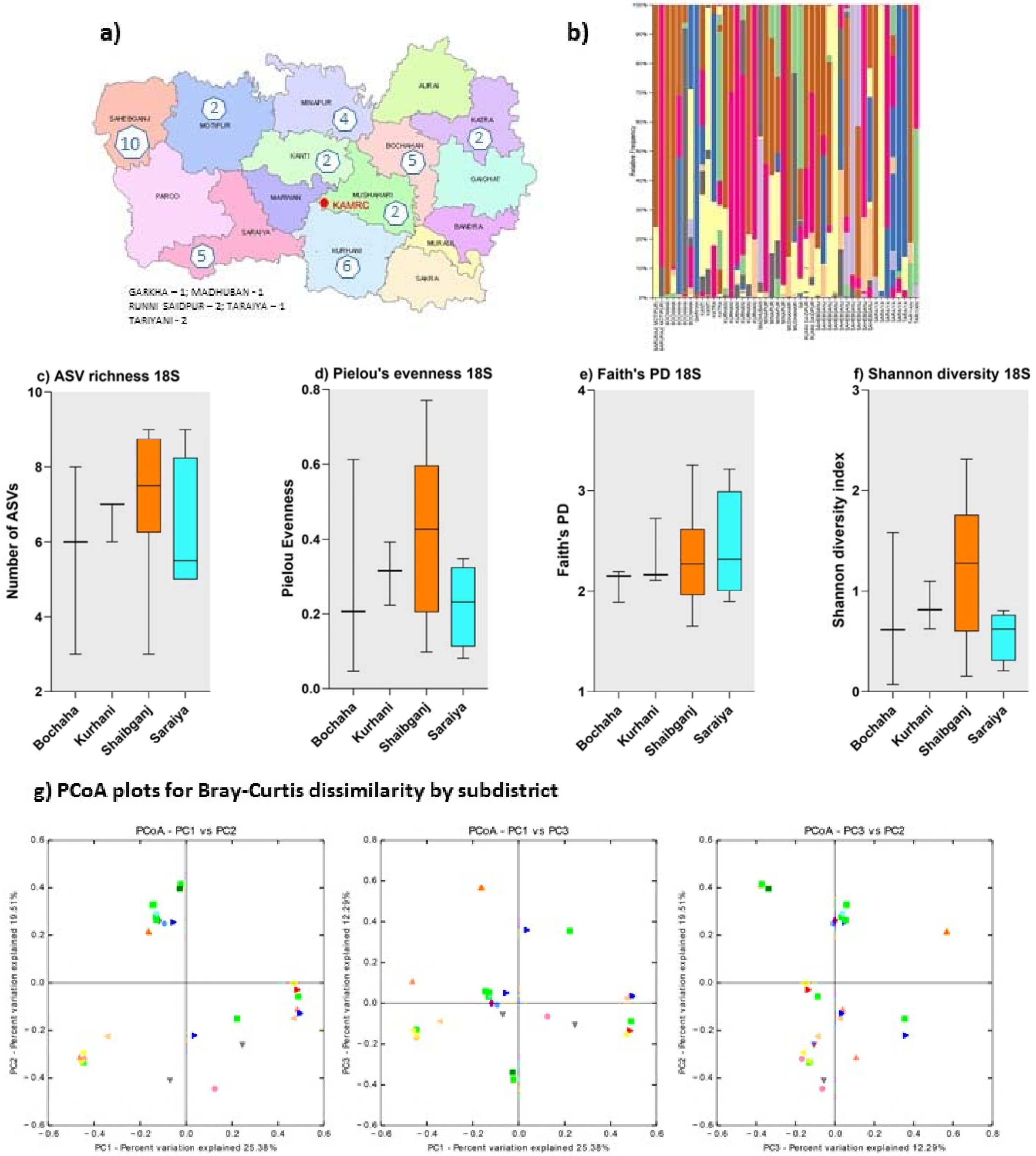

S6 Figure. Influence of micro-geography on 18S rRNA-determined prokaryotic microbial profiles. (a) map of the district of Muzaffarpur showing subdistricts or block, annotated with the numbers of samples collected from different blocks. Listed below the map are numbers of samples from blocks outside of Muzaffarpur. (b) bar plots for relative abundance of taxa by district; colour key to relative abundances of taxa as for main figure 2(a). (c) to (f) comparisons of species richness (number of ASVs) and alpha diversity measures (as labelled) by blocks with N≥3. (g) PCoA plots (left to right: PC1xPC2; PC1xPC3; PC2xPC3) for Bray's dissimilarity beta diversity by colour-coded blocks.
